# Morphology and Inheritance of Wavy Flower Form in Periwinkle (*Catharanthus roseus* (L.) G. Don)

**DOI:** 10.3390/plants13162272

**Published:** 2024-08-15

**Authors:** Ting-Hsuan Huang, Yi-Chien Lu, Yu-Huan Chen, Rong-Show Shen

**Affiliations:** 1 Department of Horticultural Science, National Chiayi University, No. 300, Xuefu Rd., East Dist., Chiayi City 600355, Taiwan; love5203550@gmail.com (T.-H.H.); elsa860622@gmail.com (Y.-C.L.); chenoscar.41111@yahoo.com.tw (Y.-H.C.); 2 School of Life Science, National Taiwan Normal University, 88 Ting-Chow Rd., Sec. 4, Taipei 116, Taiwan

**Keywords:** vinca, breeding, incomplete dominance, female sterility

## Abstract

Periwinkle (*Catharanthus roseus* (L.) G. Don) is renowned for its diverse colors and resilience to harsh climates. Still, most commercial cultivars predominantly display flat petals. Using cultivars representing non-wavy, medium-wavy, and extreme-wavy flower forms, we examined morphological differences in both their mature leaves and floral organs. Phenotypes of self-pollinated (S_1_) and cross-pollinated (F_1_, F_2_) populations further underscored their morphological distinctions. Specifically, the extreme-wavy type displayed elliptical leaves, broader than the non-wavy type, with a pronounced acute apex and a notably wrinkled blade surface. The non-wavy type also bore intensely wavy petal margins and exhibited a smaller flower diameter, with a notable absence of a functional pistil, indicating female sterility. The insights gained allowed for early differentiation during the seedling period. This study suggests that the inheritance of these flower forms is regulated by an allele *WAVY* (*Wv*), which exhibits incomplete dominance. Concretely, the non-wavy form arises from a recessive homozygous expression (*wvwv*), the extreme-wavy from a dominant homozygous expression (*WvWv*), and the medium-wavy from a heterozygous expression (*Wvwv*). This study provides clarity on morphological descriptions and inheritance patterns of wavy flower forms, facilitating strategic breeding of diverse flower forms in periwinkle.

## 1. Introduction

Periwinkle (*Catharanthus roseus* (L.) G. Don), also known as Madagascar periwinkle or vinca, belongs to the Apocynaceae family. Initially an endemic species of Madagascar, it has since been domesticated in tropical and subtropical regions due to its prolonged flowering period, diverse flower colors, resilience to heat and drought, and overall strong adaptability [1]. Presently, periwinkle ranks among the world’s most popular summer potted plants [2]. Periwinkle is enriched in terpenoid indole alkaloids, positioning it as a potential treatment for certain mammalian heart diseases and some cancerous tumors [3].

The floral structure of periwinkle is similar to that of several species within the Apocynaceae family, comprised of five flat corolla lobes and a corolla tube. The stamens and pistil are enveloped within the corolla tube [4,5]. In 2001, a double-flowered mutant, TYV1, was first discovered in the self-pollinated lines (S_1_) of the cultivar ‘Pacifica Polka Dot’ in Taoyuan City, Taiwan [6], which is also the parent for breeding the doubled-flower cultivar ‘Taoyuan No.1-Rose Girl’ [7]. By 2009, double-flowered periwinkle cultivars were introduced by Sakata Seed Co., Japan. The mutation of TYV1 was not caused by the typical transformation of stamens into petaloid organs and was recognized as a single recessive allele inheritance [6]. Companies like Suntory Flower Co. (Tokyo, Japan) have released special flower forms of periwinkles such as the ‘Soiree Kawaii’ series (mini flower forms) and the ‘Soiree Flamenco’ series (wavy flower forms). Despite these advancements, no study has reported on the description or inheritance pattern of the wavy flower form in periwinkle yet. In many other flowers, wavy petals are prevalent due to their improved aesthetic value and are often a targeted trait for breeders. Understanding the inheritance pattern of the wavy flower form can lead to more precise and efficient breeding programs. Former studies have revealed that the wavy flower form of *Primula polyantha* is controlled by an allele displaying complete dominance [8]. Unfortunately, few studies have addressed the inheritance pattern of the wavy petal phenotype, inspiring this study to investigate the morphology and inheritance pattern of periwinkles with wavy flower form.

## 2. Results

### 2.1. Morphology of Leaves of Non-Wavy, Medium-Wavy and Extreme-Wavy Types

**Figure 1:**
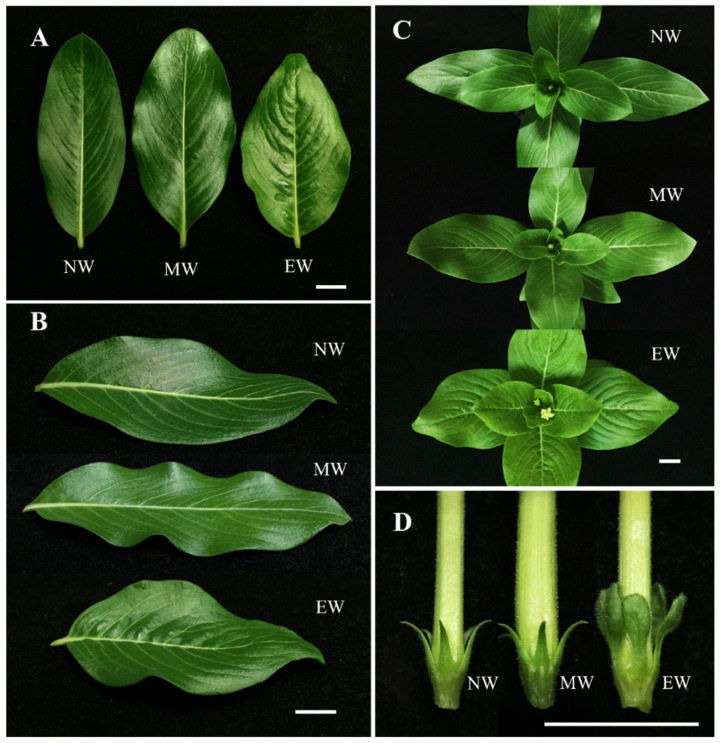
The morphology of leaves and sepals in periwinkle with non-wavy flower form (NW), medium-wavy flower form (MW), and extreme-wavy flower form (EW). (**A**) Front view of leaves; (**B**) side view of leaves; (**C**) top view of juvenile plants; (**D**) sepals. Bar = 1 cm.

Morphological differences between non-wavy type (NW), medium-wavy type (MW), and extreme-wavy type (EW) can be discerned in the leaf shape. The non-wavy type is oblong, with a smooth edge, obtuse to mucronate apex, and flat surface, while the medium-wavy type has similar leaves to the non-wavy type but a slightly wavy edge, and the extreme-wavy type exhibits an ovate shape with wider leaf width and acute apex compared to the non-wavy type (Figure 1A,B). Additionally, the leaf surface of the extreme-wavy type, particularly close to the midrib, is noticeably uneven and wrinkled, which can be discerned even in young plants (Figure 1C).

The leaf lengths of the non-wavy and medium-wavy types were significantly longer than that of the extreme-wavy type, and the widths of the medium-wavy and extreme-wavy types were significantly wider than that of the non-wavy type (Table 1). For the length:width ratio, the non-wavy type had the highest ratio, followed by the medium-wavy type and extreme-wavy type, a result consistent with observed leaf appearances, indicating an obvious ovate shape.

### 2.2. Morphology of Flowers of Non-Wavy, Medium-Wavy, and Extreme-Wavy Types

**Figure 2:**
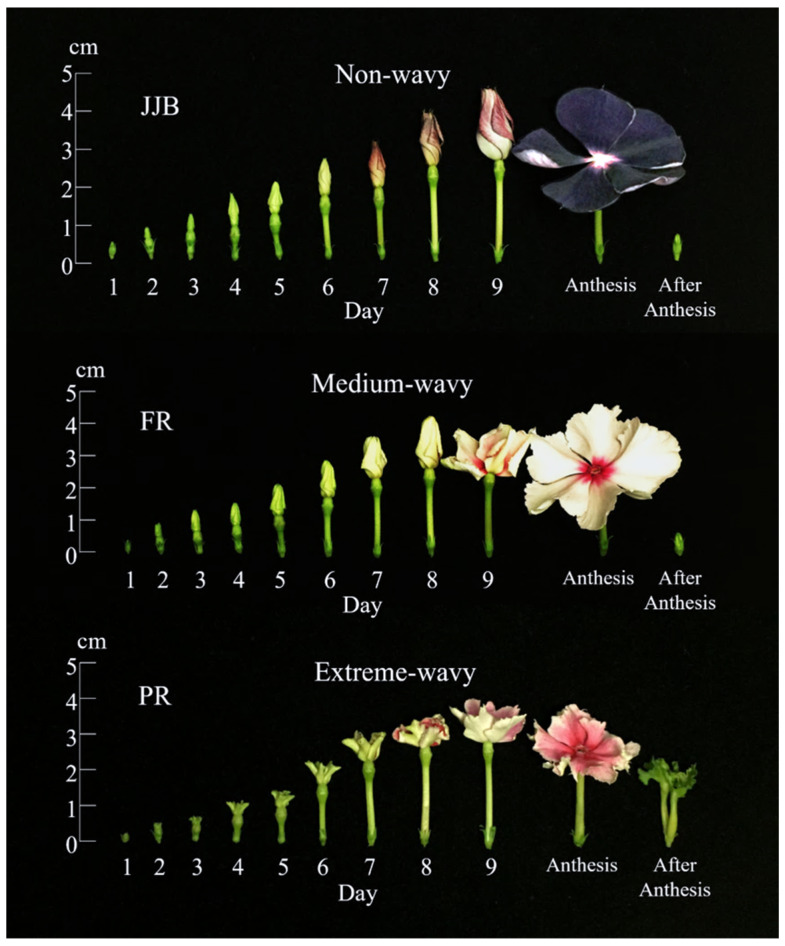
The development of flowers of periwinkle of the non-wavy, medium-wavy, and extreme-wavy flower forms. The appearance from 0.5 cm flower buds to anthesis and the residuals after withering; JJB = Jams ‘N Jellies Blackberry; FR = Furau; PR = Purinsesu Chuchu Reddo.

The sepal apices of non-wavy and medium-wavy types were both narrowly acuminate, while that of the extreme-wavy type was occasionally obtuse to truncate (Figure 1D). The floral organ development of the non-wavy, medium-wavy, and extreme-wavy types did not show significant differences in terms of timing (Table 2), but there were considerable differences in petal appearance (Figure 2). During the later development period (days 6–8 of flower development), the petal arrangements of both the medium-wavy and extreme-wavy types were loose, overlapping, and uneven. Particularly, the extreme-wavy type had noticeably unsmooth petals even in the early period. Conversely, the petals of the non-wavy type only loosened up the day before blooming, exhibiting a neat and flat arrangement.

**Figure 3:**
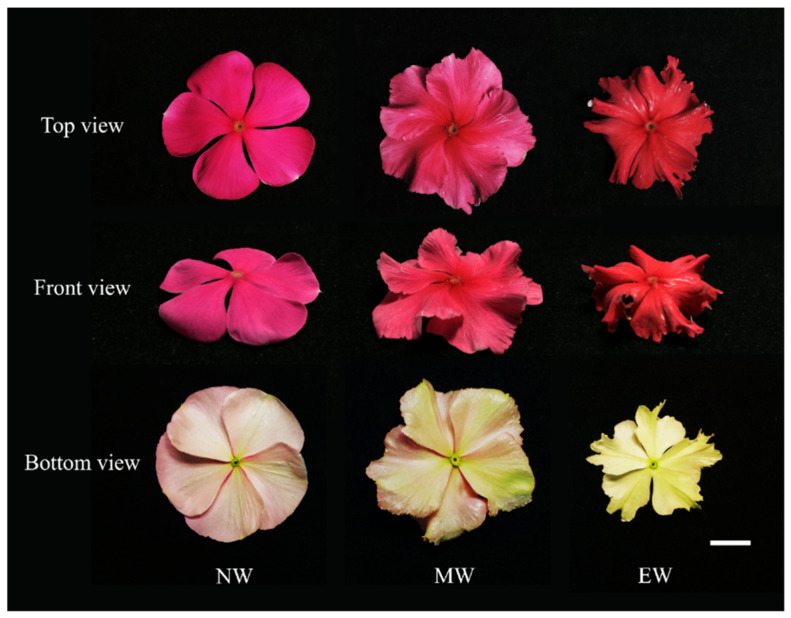
Flower top, front, and bottom views of periwinkle of the non-wavy, medium-wavy, and extreme-wavy flower forms; NW = non-wavy; MW = medium-wavy; EW = extreme-wavy; Bar = 1 cm.

The most distinct differences among the non-wavy, medium-wavy, and extreme-wavy types were found in the waviness of the petals and the size of the flower diameter. Non-wavy types had flat petals with smooth margins, and their abaxial sides were predominantly white (Figure 3). Medium-wavy-type petals appeared wavy when viewed from the front, the margins were unsmooth and slightly jagged, and the abaxial sides were also white, but with deeper green spreading from the corolla tube towards the outer margin, and irregular spikes were observed on the margin and abaxial side. Extreme-wavy-type petals appeared wavy as well, with more pronounced and irregular jaggedness at the margin compared to the medium-wavy type, and the abaxial side exhibited deeper and more prominent green spreading than the non-wavy and medium-wavy types.

**Figure 4:**
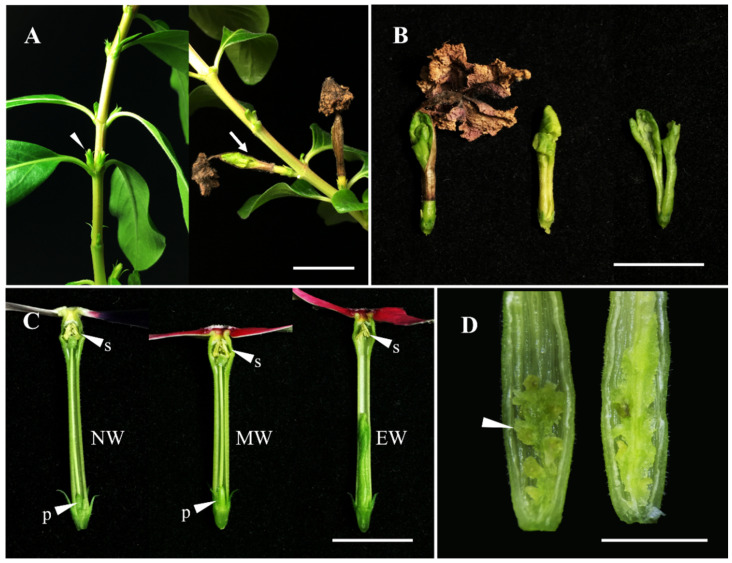
Morphology and anatomy of periwinkle of the non-wavy, medium-wavy, and extreme-wavy flower forms. (**A**) Unpollinated flower organs after flower withering of non-wavy (left; arrow) and medium-wavy (right; arrow) types. (**B**) The wilted extreme-wavy flower stuck to the abnormally developed pistil consisting of two closely intertwined blades. (**C**) The non-wavy and the medium-wavy flowers had normal stamens (s) and pistils (p), while the extreme-wavy flowers had normal stamens but lacked normal pistils (arrow). (**D**) Several placental-like and ovule-like (arrowheads) organs at the base of the extreme-wavy flower. NW = non-wavy; MW = medium-wavy; EW = extreme-wavy. Bar = 1 cm.

After flower wilting, the flowers of both the non-wavy and medium-wavy types fell off naturally, leaving behind the ovary and calyx on the plant. In contrast, the wilted flowers of the extreme-wavy type stuck to the plant due to the abnormal growth of the pistils (Figure 4A,B). In the cross-section of the floral organs, the stamens and pistils of the non-wavy and medium-wavy types were normal and fertile, while the stamens of the extreme-wavy type were fertile as well (Figure 4C); However, its pistils were abnormally developed (Figure 4A), consisting of two closely intertwined blades with the middle and bottom segments rolling inward (Figure 4B); several ovule-like and placenta-like organs located at the bottom segment (Figure 4D); and the end segment unfolded, presenting an irregular shape where the two structures tightly wrapped each other (Figure 4B).

The flower diameter of the non-wavy type was significantly the largest, followed by the medium-wavy type and the extreme-wavy type (Table 1). The wave height at the petal margins of the medium-wavy type was the greatest, followed by the extreme-wavy type, and the petals of non-wavy type were flat, lacking significant waves. Regarding petal weight, the medium-wavy type had the highest fresh and dry weight of petals, and there were no significant differences between the fresh and dry weight of the petals for the non-wavy and extreme-wavy types (Table 1).

### 2.3. Inheritance of Non-Wavy, Medium-Wavy, and Extreme-Wavy Types

Seven commercial cultivars of periwinkle were selected for this study: five non-wavy types (NW), namely, ‘Natsu Sakura’ (Summer Cherry Blossom, SS), ‘Jams ‘N Jellies Blackberry’ (JJB), ‘Mediterranean XP Rose Halo’ (MRH), ‘Tattoo Papaya’ (TP), and ‘Tattoo Blackberry’ (TB); one medium-wavy type (MW) with slightly wavy petals, ‘Furau’ (Frau, FR); and one extreme-wavy type (EW) with intensely wavy petals, ‘Purinsesu Chuchu Reddo’ (Princess Tutu Red, PR).

The petal waviness of the self-pollinated lines (S_1_) of non-wavy-type parents (SS, JJB, MRH, TP, and TB) did not segregate and only produced non-wavy-type offspring (Table 3), indicating that these parents were possibly homozygous. On the other hand, the petal waviness of the S_1_ generation of medium-wavy-type parents (FR) showed segregation. A chi-square test was performed (*p* = 0.136; χ^2^ = 3.987), and the tested group had *p* > 0.05, indicating no significance difference between the observed ratio and the expected ratio, accepting the ratio of 1:2:1 (Table 3). This suggested that medium-wavy-type parents (FR) was heterozygous, and the medium-wavy-type and extreme-wavy-type flower forms were likely related.

In the F_1_ generation produced by cross-pollination between the non-wavy type and medium-wavy type (SS × FR, JJB × FR, TP × FR, and TB × FR), segregated phenotypes of the non-wavy type and medium-wavy type were observed, but the extreme-wavy type was not present. The chi-square test was performed on all cross-pollinated combinations, and all had *p* > 0.05, implying that the groups satisfied the 1:1 ratio (Table 3).

**Figure 5:**
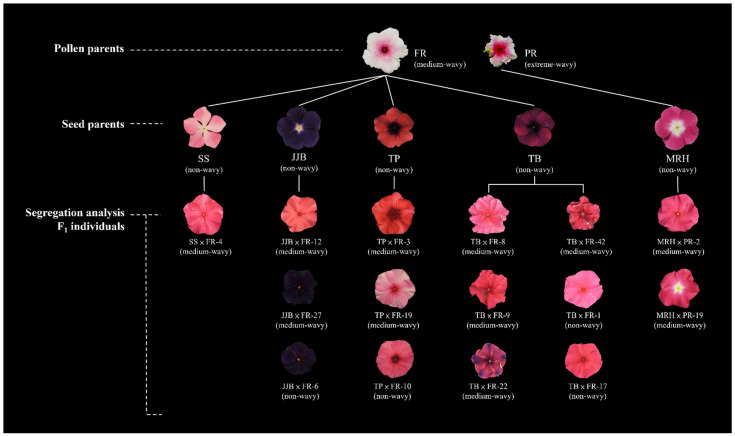
Top view of flowers of the pollen parents, seed parents, and F_1_ individuals selected for F_2_ generation wavy flower form segregation analysis.

In the F_1_ population, a few individuals were randomly selected from different combinations: SS × FR-4 (MW) from SS × FR; JJB × FR-12 (MW), JJB × FR-27 (MW), and JJB × FR-6 (NW) from JJB × FR; TP × FR-3 (MW), TP × FR-19 (MW), and TP × FR-10 (NW) from TP × FR; and TB × FR-8 (MW), TB × FR-9 (MW), TB × FR-22 (MW), TB × FR-42 (MW), TB × FR-1 (NW) and TB × FR-17 (NW) from TP × FR (Figure 5).

**Figure 6:**
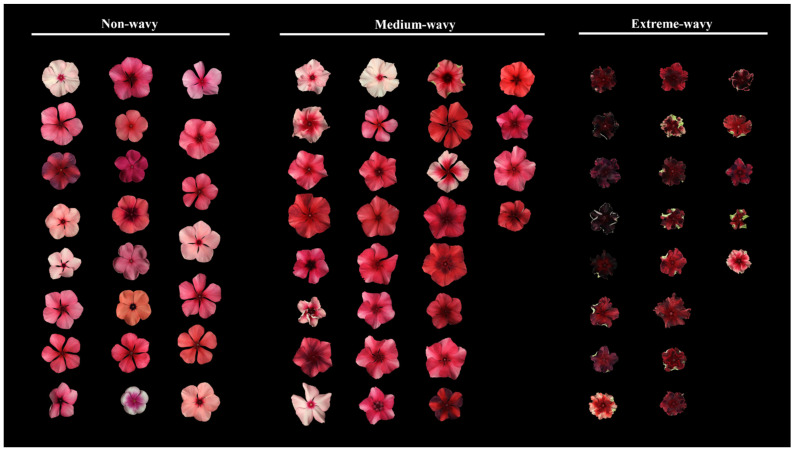
Segregation of non-wavy, medium-wavy, and extreme-wavy flower forms in the F_2_ generation of F_1_ line TP × FR-3.

The non-wavy type F_1_ individuals were self-pollinated, and the F_2_ populations were all non-wavy-type. In contrast, all nine F_2_ populations of medium-wavy-type F_1_ were segregated into not only the non-wavy type and medium-wavy type, but also the extreme-wavy type. All the chi-square tests returned *p* > 0.05, indicating that they satisfied the 1:2:1 ratio (Table 3). This is similar to the medium-wavy-type parent (FR) self-pollination line. For instance, the F_2_ of TP × FR-3 consisted of 72 individuals, further classified into 23 non-wavy, 28 medium-wavy, and 21 extreme-wavy types (Figure 6). The chi-square test for this group gave a χ^2^ value of 3.667 and a *p* value of 0.16, meeting the expected 1:2:1 ratio (Table 3).

For the F_1_ generation from cross-pollination between the non-wavy type and extreme-wavy type (MRH × PR), all 87 plants were of the medium-wavy type. In the F_1_ population, two medium-wavy-type individuals, MRH × PR-2 and MRH × PR-19, were selected. MRH × PR-2 and MRH × PR-19 were self-pollinated, and the F_2_ generation of MRH × PR-2 segregated into 22 non-wavy-type, 34 medium-wavy-type, and 15 extreme-wavy-type individuals. The chi-square test for this group resulted in χ^2^ = 1.507; *p* = 0.471. Similarly, F_2_ of MRH × PR-19 segregated into 13 non-wavy-type, 49 medium-wavy-type, and 25 extreme-wavy-type individuals. The chi-square test returned χ^2^ = 4.701; *p* = 0.095. Since all tests returned *p* > 0.05, it was deemed that they satisfied the 1:2:1 ratio (Table 3).

## 3. Discussion

Commercial periwinkle cultivars often lack morphological diversity, particularly with respect to their flower forms. Most of these cultivars possess non-wavy-type petals, which can diminish their novelty. In this context, introducing medium-wavy and extreme-wavy types could increase diversity in periwinkle flower forms. This study provides insights into the morphological differences and inheritance patterns of these traits.

Non-wavy, medium-wavy, and extreme-wavy types exhibit significant differences in petal wave heights and flower diameters. In the early stages of extreme-wavy-type seedlings, characteristics like leaf and calyx features can be used for their early selection and identification. Identifying medium-wavy types among seedlings can be challenging, as their leaf edges, although somewhat wavy, are not easily distinguishable from non-wavy types during the seedling stage. However, observing the bud stage for early petal loosening, a characteristic of medium-wavy types, can assist in their early identification. With these selection strategies, breeders can accelerate the process of wavy-flower periwinkle breeding.

Very few studies have delved into the inheritance of wavy flower forms. Existing research has generally determined that the inheritance of these forms is controlled by a single dominant gene. In this study, all progeny in the S_1_ generation of the non-wavy-type parents displayed the non-wavy type, confirming homozygosity and the absence of genes responsible for medium-wavy or extreme-wavy types. Conversely, the self-pollinated line of the medium-wavy-type parent, FR, produced offspring exhibiting non-wavy, medium-wavy, and extreme-wavy types. Moreover, all plants in the F_1_ generation resulting from cross-pollination between non-wavy and extreme-wavy types were of the medium-wavy type. From the observations of this study, the medium-wavy and extreme-wavy types might be controlled by the same gene, with FR being heterozygous and PR being homozygous for this gene.

Cross-pollination of the non-wavy and medium-wavy types in the F_1_ generation only yielded non-wavy and medium-wavy phenotypes, adhering to a 1:1 ratio. Self-pollination of the selected medium-wavy type individuals produced a segregation ratio matching that of the FR self-pollination line, following a 1:2:1 ratio for non-wavy, medium-wavy, and extreme-wavy types. In contrast, self-pollination of the selected non-wavy type individuals produced only non-wavy-type offspring. The cross-pollinated F_2_ offspring of the non-wavy and extreme-wavy types exhibited the same pattern as the FR self-pollination line. These findings affirm that the medium-wavy and extreme-wavy types are controlled by an intermediate dominant allele. This study corroborates that the wavy flower form in periwinkle is governed by the *WAVY* allele. When the allele is homozygous dominant (*WvWv*), the resulting phenotype is the extreme-wavy type; when it is heterozygous (*Wvwv*), the phenotype is the medium-wavy type; and when it is homozygous recessive (*wvwv*), the phenotype is the non-wavy type.

Interestingly, the extreme-wavy type is characterized by an abnormal pistil structure, which leads to female sterility and premature flower withering. This abnormality might be due to either the linkage with the wavy petal trait or the malfunction of *WAVY* related to pistil development, as suggested by previous breeding studies [9,10]. Alternatively, *WAVY* might display pleiotropy, affecting the development of multiple organs simultaneously, and influence leaf development, leading to wider, wrinkled leaves. This trait is most pronounced in the extreme-wavy type. Out of all periwinkles bred in this study, only the extreme-wavy type exhibited phenotypes of wider leaves, unevenly wrinkled leaf surfaces, and abnormal pistils. This observation leads us to infer that gene linkage is less likely to be responsible for these traits. Instead, pleiotropy seems to be a more plausible explanation for these observed phenomena.

For the non-wavy type, the wavy flower form emerges as a dominant trait, mirroring the inheritance patterns observed in *Primula polyantha* [8]. The creased petal phenotype strain Q467 in Japanese morning glory (*Ipomoea nil*) was acquired by crossing TKS with a *fe* mutant strain (Q441) with the transposon-inserted *FEATHERED* (*FE*) gene, which caused severe curling and deformities in the leaves. This suggests that the dysfunction of the *FE* gene impacted the abaxial identity of organ cell differentiation [11]. Another investigation into Japanese morning glory revealed that the *maple–willow* mutant’s petals became choripetalous, and its leaf wrinkles resembled those of wavy-petal-form periwinkles, with its pistil also developing abnormally [12,13].

Prior research has indicated that the *TEOSINTE BRANCHED1, CYCLOIDEA, and PCF* (*TCP*) family influence the development of leaves and petals. For instance, the *tcp* mutant petal of *Arabidopsis thaliana* was reported to be wavy, with mutant cells at the petal edges being elliptical, unlike the round cells of the wild type [14]. In this study, we used optical microscopes to observe a similar pattern in the flower forms of the medium-wavy and extreme-wavy types. In roses (*Rosa hybrida*), when the function of the *TCP3* gene was knocked down, the flower diameter decreased, the petal edges became jagged, the leaves wrinkled and broadened, and the calyx took on leaf-like qualities [15]. These were similar to the effect of the extreme-wavy type in the present study. While no research has delved into the *TCP* transcription factor in periwinkles, a microRNA study found that periwinkles harbor three *miR319* that inhibit *TCP* transcription [16]. Consequently, we speculate that periwinkle does possess the *TCP* transcription factor, and the wavy flower trait may arise from a loss of function in the *TCP* transcription factor, leading to a unique flower shape.

## 4. Materials and Methods

### 4.1. Plant Materials

All non-wavy-type cultivars (SS, JJB, MRH, TP, and TB) were cultivated from purchased F_1_ hybrid seeds, whereas the medium-wavy-type (FR) and extreme-wavy-type (PR) cultivars were clonal cultivars obtained as healthy plants from the local market. In November 2019, all parental seeds were sown in a 72-hole seedling tray using a 1:1 (*v*/*v*) mixture of peat and perlite as the growing medium. The seedlings were cultivated on a growing bench with a flood irrigation system in the National Chiayi University greenhouse in Taiwan. The mean daily light intensity at noon was 1011 μmol·m^−2^·s^−1^. The mean daytime and nighttime temperatures were 32 °C and 26 °C, respectively, in summer, and 26 °C and 18 °C, respectively, in winter. Plants were fertilized weekly with water-soluble 20N–8.7P–16.6K fertilizer (Hyponex Plant Food 20-20-20, Hyponex Co., Maryville, OH, USA) at 1 g·L^−1^ and watered as required. Once the seedlings developed three to four pairs of fully extended true leaves, they were transplanted to 9 cm diameter plastic pots. The medium and cultivation methods used were identical to those previously described.

### 4.2. Morphological Investigation of Non-Wavy, Medium-Wavy, and Extreme-Wavy Types

Medium-wavy-type and extreme-wavy-type cultivars were represented by FR and PR, respectively. For observation of the leaves’ morphology, ten mature leaves from each cultivar were measured for their length and width with a digital caliper (0.01 mm resolution). For floral organ development, ten 0.5 cm young flower buds from each cultivar were marked, with the surrounding buds removed. Bud developments were measured daily at 17:00 with a digital caliper until the flowers fully opened. For the anatomical observation of the floral organ, flowers that fully opened on the first day were selected and cut open vertically, and the calyx, stamens, and pistil were observed with a stereomicroscope.

### 4.3. Inheritance of Non-Wavy, Medium-Wavy, and Extreme-Wavy Types

Autogamy occasionally occurs in the periwinkle bud stage, necessitating emasculation and artificial pollination for cross-pollination. This study employed the pollination method described by a previous study [5]. Emasculation was performed two days before anthesis by removing about 1.5 cm of the corolla tube with a scalpel, and paternal pollen was collected using a 5 cm fishing string to artificially apply to maternal stigma. The parental cultivars SS, JJB, MRH, TP, TB, and FR were self-pollinated and cross-pollinated, except for the extreme-wavy-type cultivar PR, which lacked a fertile pistil and therefore could not be self-pollinated. The medium-wavy type (FR) was used as the pollen parent to cross-pollinate non-wavy types (SS, JJB, TP, and TB) to generate the F_1_ hybrid generation. Likewise, the extreme-wavy type (PR) was crossed with the non-wavy type (MRH).

To investigate the segregation ratio, F_1_ was self-pollinated to produce F_2_, the trait-segregated generation. This generated six parent self-pollination lines (S_1_), five F_1_, and eight F_2_. In January 2020, 200 seeds were sown for each S_1_ and F_1_. In March 2021, approximately 30 to 200 seeds were sown for each F_2_. Cultivation methods and environmental conditions were consistent with those mentioned above. Upon blooming of the plant’s second flower, individual petal phenotypes of each population were recorded. A chi-square test was employed to calculate the χ^2^ value of the segregation ratio of the offspring flower form segregation to compare the actual ratios with the expected ratios for significance of fit.

### 4.4. Statistics

The experiment adopted a complete randomized design. Statistical software Costat 6.4 (Cohort software, Monterey, CA, USA) was used for data analysis, namely, analysis of variance and calculation of least significant difference to determine whether the quantitative traits of each cultivar exhibited significant differences at *p* < 0.05. For the inheritance pattern test, the segregation ratio was calculated and chi-square analysis was used to calculate χ^2^ and the probability. When *p* > 0.05, the test segregation ratio was accepted.

## 5. Conclusions

The novel medium-wavy and extreme-wavy flower types of periwinkle (*Catharanthus roseus*) are regulated by the *WAVY* gene, which exhibits incomplete dominance. Analysis of F_1_, F_2_, and self-pollinated parental populations demonstrated that the common flat flower type (non-wavy) is regulated by the homozygous recessive genotype, while the extreme-wavy type corresponds to the homozygous dominant genotype. The medium-wavy phenotype is associated with the heterozygous genotype. This study provides a foundational framework for breeders to design crossing strategies aimed at generating a broader range of flower forms, thereby enhancing the diversity available to consumers. Additionally, for breeding wavy-flower periwinkle, breeders can predict the ratio of desired flower types in the offspring based on the genotypes of the parents. The distinct characteristics of extreme-wavy plants, such as notably wide leaves with abnormal wrinkles, an acute leaf apex, and a loose bud structure, also enable early identification and selection during the breeding process.

## Figures and Tables

**Table 1:** Morphological survey of leaves and flowers of periwinkle of the non-wavy, medium-wavy, and extreme-wavy flower forms.

Phenotype (Cultivar) ^1^	Leaf			Flower				
	Length (cm)	Width (cm)	Length:Width Ratio ^3^	Diameter (cm)	Wave Height of Petal (cm)	Fresh Weight of Corolla (g)	Dry Weight of Corolla (g)	Diameter:Fresh Weight Ratio ^4^
NW (JJB)	7.39 a ^2^	3.05 b	2.44 a	4.77 a	0.36 c	0.23 b	0.3 b	21.6 a
MW (FR)	7.46 a	3.56 a	2.11 b	4.27 b	1.21 a	0.27 a	0.41 a	16.16 b
EW (PR)	6.45 b	3.74 a	1.73 c	3.23 c	1.02 b	0.21 b	0.25 b	17.11 b

^1^ NW = non-wavy; MW = medium-wavy; EW = extreme-wavy. JJB = Jams ‘N Jellies Blackberry; FR = Furau; PR = Purinsesu Chuchu Reddo. ^2^ Means within each column followed by different letter(s) are significantly different at *p* < 0.05 according to Fisher’s LSD test. ^3^ Means are averaged from the ratio of each examined leaf. ^4^ Means are averaged from the ratio of each examined corolla.

**Table 2:** The daily development and growth of flower buds from the length of 0.5 cm to flowering of periwinkle of the non-wavy, medium-wavy, and extreme-wavy flower forms.

Phenotype	Day								
(Cultivar) ^1^	1	2	3	4	5	6	7	8	9
	Length of flower bud (cm)								
NW (JJB)	0.52	0.57	0.80	0.97	1.25	1.71	2.16	3.06	4.38
MW (FR)	0.50	0.61	0.87	1.08	1.49	2.21	2.73	3.92	4.40
EW (PR)	0.56	0.60	0.83	0.97	1.08	1.54	1.89	2.45	3.23
	Relative growth (cm)								
NW (JJB)	-	0.05	0.24	0.16	0.28	0.46	0.45	0.90	1.33
MW (FR)	-	0.11	0.27	0.21	0.41	0.72	0.52	1.18	0.48
EW (PR)	-	0.05	0.23	0.13	0.11	0.46	0.34	0.56	0.78

^1^ NW = non-wavy; MW = medium-wavy; EW = extreme-wavy. JJB = Jams ‘N Jellies Blackberry; FR = Furau; PR = Purinsesu Chuchu Reddo.

**Table 3:** Segregation of non-wavy, medium-wavy, and extreme-wavy flower forms in progenies of self- and cross-pollinated periwinkle cultivars.

Parents/Crosses/Generation		Total	Flower Form			Test Ratio	χ^2^	*p* ^x^
			NW	MW	EW			
Cultivar (flower form)								
	FR⊗ (MW)	77	25	30	22	1:2:1	3.987	0.136
	PR⊗ (EW)	- ^z^	-	-	-	-	-	
	SS⊗ (NW)	200	200	0	0	1:0:0	all NW	
	JJB⊗ (NW)	175	175	0	0	1:0:0	all NW	
	MRH⊗ (NW)	158	158	0	0	1:0:0	all NW	
	TP⊗ (NW)	100	100	0	0	1:0:0	all NW	
	TB⊗ (NW)	136	136	0	0	1:0:0	all NW	
F_1_								
	SS (NW) × FR (MW)	70	33	37	0	1:1:0	0.229	0.633
	JJB (NW) × FR (MW)	93	49	44	0	1:1:0	0.269	0.604
	TP (NW) × FR (MW)	45	28	17	0	1:1:0	2.689	0.101
	TB (NW) × FR (MW)	60	37	23	0	1:1:0	3.267	0.071
	MRH (NW) × PR (EW)	87	0	87	0	0:1:0	all MW	
F_2_								
	SS × FR-4 ^y^⊗ (MW)	101	23	50	28	1:2:1	0.505	0.777
	JJB × FR-12⊗ (MW)	15	4	9	2	1:2:1	1.133	0.567
	JJB × FR-27⊗ (MW)	26	7	16	3	1:2:1	2.615	0.270
	TP × FR-3⊗ (MW)	72	23	28	21	1:2:1	3.667	0.160
	TP × FR-19⊗ (MW)	32	6	20	6	1:2:1	2.000	0.368
	TB × FR-8⊗ (MW)	67	11	37	19	1:2:1	2.642	0.267
	TB × FR-9⊗ (MW)	51	13	22	16	1:2:1	1.314	0.518
	TB × FR-22⊗ (MW)	51	10	25	16	1:2:1	1.431	0.489
	TB × FR-42⊗ (MW)	87	18	53	16	1:2:1	4.241	0.120
	MRH × PR-2⊗ (MW)	71	22	34	15	1:2:1	1.507	0.471
	MRH × PR-19⊗ (MW)	87	13	49	25	1:2:1	4.701	0.095
	JJB × FR-6⊗ (NW)	59	59	0	0	1:0:0	all NW	
	TP × FR-10⊗ (NW)	70	67	0	0	1:0:0	all NW	
	TB × FR-1⊗ (NW)	126	126	0	0	1:0:0	all NW	
	TB × FR-17⊗ (NW)	35	35	0	0	1:0:0	all NW	

^z^ Extreme-wavy flower form phenotype is unable to self-pollinate due to abnormal pistil. ^y^ Code number of selected F_1_ progeny. ^x^ Chi-square analysis shows *p* > 0.05, indicating that the observed data conform to the expected ratio. FR = Furau; PR = Purinsesu Chuchu Reddo; SS = Natsu Sakura; JJB = Jams ‘N Jellies Blackberry; MRH = Mediterranean XP Rose Halo; TP = Tattoo Papaya; TB = Tattoo Blackberry; NW = non-wavy; MW = medium-wavy; EW = extreme-wavy.

## Data Availability

The data generated in this study are included in this published article.

## References

[1] Levy A., Halevy A.H. (1981). Catharanthus roseus. CRC Handbook of Flowering.

[2] Curry H.A. (2012). Naturalising the exotic and exoticising the naturalised: Horticulture, natural history and the rosy periwinkle. Environ. Hist..

[3] Zhou M.L., Shao J.R., Tang Y.X. (2009). Production and metabolic engineering of terpenoid indole alkaloids in cell cultures of the medicinal plant *Catharanthus roseus* (L.) G. Don (Madagascar periwinkle). Biotechnol. Appl. Biochem..

[4] van Bergen M.A. (1996). Revision of *Catharanthus* G. Don. series of revisions of Apocynaceae–XLI. Wagening. Agric. Univ. Pap..

[5] Miyajima D. (2004). Pollination and seed set in vinca [*Catharanthus roseus* (L.) G. Don]. J. Hortic. Sci. Biotechnol..

[6] Chen C.M., Wei T.Y., Yeh D.M. (2012). Morphology and inheritance of double floweredness in *Catharanthus roseus*. HortScience.

[7] Chen C.M., Yeh D.M. (2012). ‘Taoyuan No. 1 Rose Girl’: A double-flowered periwinkle, *Catharanthus roseus*. HortScience.

[8] Song C. (2006). Inheritance of qualitative character in *Primula polyantha*. Korean J. Hortic. Sci. Technol..

[9] Lin H.K. (2016). Breeding of Double-Flowered and Trailing *Catharanthus roseus*. Master’s Thesis.

[10] Tsai Y.T. (2018). Male Sterility, Inheritance of Flower Color, and Selection of Potted Plants in Periwinkle. Master’s Thesis.

[11] Iwasaki M., Nitasaka E. (2006). The *FEATHERED* gene requirement in lateral organs polarity establishment, especially flowers of the Japanese morning glory (*Ipomoea nil*). Plant Mol. Biol..

[12] Kajita Y., Nishino E. (2009). Leaves and flowers development in the wild type and pleiotropic *maple-willow* mutant of Japanese morning glory (*Ipomoea nil*). J. Jpn. Soc. Hortic. Sci..

[13] Kajita Y., Nishino E. (2009). Morphology and anatomy of leaves and flowers of wild-type and pleiotropic *maple-willow* mutant in Japanese morning glory (*Ipomoea nil*). J. Jpn. Soc. Hortic. Sci..

[14] Koyama T., Ohme-Takagi M., Sato F. (2011). Generation of serrated and wavy petals by inhibition of the activity of *TCP* transcription factors in *Arabidopsis thaliana*. Plant Signal. Behav..

[15] Gion K., Suzuri R., Shikata M., Mitsuda N., Oshima Y., Koyama T., Ohme-Takagi M., Ohtsubo N., Tanaka Y. (2011). Morphological changes of *Rosa × hybrida* by a chimeric repressor of *Arabidopsis* TCP3. Plant Biotechnol..

[16] Shen E.M., Singh S.K., Ghosh J.S., Patra B., Paul P., Yuan L., Pattanaik S. (2017). The miRNAome of *Catharanthus roseus*: Identification, expression analysis, and potential roles of microRNAs in regulation of terpenoid indole alkaloid biosynthesis. Sci. Rep..

